# The Chlamydia trachomatis Plasmid and CT135 Virulence Factors Are Not Essential for Genital Tract Infection or Pathology in Female Pig-Tailed Macaques

**DOI:** 10.1128/IAI.00121-18

**Published:** 2018-04-23

**Authors:** Dorothy L. Patton, Yvonne C. Sweeney, Audrey E. Baldessari, Linda Cles, Laszlo Kari, Gail L. Sturdevant, Chunfu Yang, Harlan D. Caldwell

**Affiliations:** a Department of Obstetrics and Gynecology, University of Washington, Seattle, Washington, USA; b Washington National Primate Research Center, University of Washington, Seattle, Washington, USA; c Chlamydia Reference Laboratory, University of Washington, Seattle, Washington, USA; d Laboratory of Bacteriology, Rocky Mountain Laboratories, National Institute of Allergy and Infectious Diseases, Hamilton, Montana, USA; e Laboratory of Virology, National Institute of Allergy and Infectious Diseases, Hamilton, Montana, USA; f Laboratory of Clinical Immunology and Microbiology, National Institute of Allergy and Infectious Diseases, Bethesda, Maryland, USA; Washington State University

**Keywords:** Chlamydia, macaques, mutants, pathogenesis

## Abstract

The Chlamydia trachomatis plasmid and inclusion membrane protein CT135 are virulence factors in the pathogenesis of murine female genital tract infection. To determine if these virulence factors play a similar role in female nonhuman primates, we infected pig-tailed macaques with the same C. trachomatis strains shown to be important in the murine model. Wild-type C. trachomatis and its isogenic mutant strain deficient in both plasmid and CT135 were used to infect macaques. Macaques were given primary and repeated cervicovaginal challenges with the wild-type and mutant strains. The infection rate, infection duration, and antibody response were similar among macaques infected with both strains. Unexpectedly, colposcopy, laparoscopy, and histologic analysis revealed no substantial genital tract pathology following either primary or repeated cervicovaginal challenges. Cytokine analysis of cervicovaginal secretions from both challenged groups revealed low concentrations of interleukin 1β (IL-1β) and elevated levels of the interleukin 1 receptor agonist (IL-1RA). We propose that an imbalance of IL-1β and IL-1RA in macaques is the reason for the mild inflammatory responses observed in infected urogenital tissues. Thus, understanding the pathobiology of chlamydial infection requires a better understanding of host epigenetic and chlamydial genetic factors. Our findings also have implications for understanding the high frequency of asymptomatic infections in humans.

## INTRODUCTION

The global prevalence of sexually transmitted infections (STIs) represents a significant public health burden. Chlamydia trachomatis is a mucosotropic obligate intracellular bacterium of epithelial cells. It is the most common cause of bacterial STI and is the etiological agent of trachoma, the leading cause of infectious preventable blindness worldwide. C. trachomatis is responsible for millions of genital tract infections in women in the United States, and the World Health Organization estimated that 131 million new cases of genital chlamydial infections occurred globally in 2016 (1). In the United States, about 1.3 million new cases of genital chlamydial infections occur annually in women ages 15 to 39 (2), at an estimated annual cost of $520 million dollars (3). Chlamydial infection and its sequelae disproportionately affect young women, with cervical infections frequently ascending to the upper reproductive tract (URT). Chlamydial infections are associated with a vast range of morbidity, from asymptomatic, self-limited infections to pelvic inflammatory disease and tubal factor infertility. Human studies of the long-term sequelae of chlamydial URT infection, including tubal fibrosis and adhesions, are limited. Importantly, chlamydial infection is also known to significantly increase the risk of HIV acquisition (4–6). Current control strategies include improved diagnostic and screening procedures for intervention and education. Unfortunately, these strategies are not highly effective; therefore, research efforts are needed to develop multipurpose prevention strategies, including the development of topical microbicides and vaccines, for the control and prevention of C. trachomatis genital infection. Scientific advances in preclinical animal models provide renewed optimism for vaccine development (7); however, to date, a vaccine has not been approved for use in humans.

Recent studies have shown that plasmid-free chlamydiae are attenuated in both mouse and nonhuman primate infection models (8–10), findings that provide optimism about the development of live-attenuated antichlamydial vaccines. O'Connell et al. (10) first described that a plasmid-deficient Chlamydia muridarum strain, a mouse-specific pathogen, was partially attenuated for the mouse female genital tract and that infection provided partial protection against rechallenge with plasmid-positive organisms. Carlson et al. (8) similarly showed that the 50% infectious dose (ID_50_) of a plasmid-deficient C. trachomatis LGV human strain was 400 times greater than that of its plasmid positive parenteral strain. Olivares-Zavaleta et al. (11) reported that cervicovaginal infection with the plasmid-deficient LGV strain provided partial protection against virulent plasmid-positive organisms. These early studies provided the impetus to use plasmid-deficient organisms as live-attenuated chlamydial STD vaccines. Most encouraging toward this goal was the finding of Kari et al. (9) that a plasmid-free C. trachomatis strain was highly attenuated for the eye in ocular infection of nonhuman primates and provided a significant level of protective immunity to challenge with virulent C. trachomatis organisms. A caveat in those studies, however, is that the plasmidless C. trachomatis strain used has chromosomal gene mutations that involved disruption of the inclusion membrane protein CT135 (12), the CT166 cytotoxin (13), and the tryptophan synthase operon (14, 15), all of which might be important virulence factors. Qu et al. (16) recently used a rhesus macaque female genital tract model to investigate whether a plasmid-deficient C. trachomatis urogenital strain might serve as a live-attenuated vaccine for chlamydial STD. Their findings were both surprising and contradictory to previous studies with plasmid-deficient strains in the mouse genital tract and nonhuman primate trachoma models. They reported that infection with either the serovar D wild-type plasmid-positive strain or a plasmidless mutant failed to produce significant pathology in the rhesus macaque model. They concluded that the presence or absence of the plasmid played no role in determining the outcome of infection.

Sturdevant et al. (17) have shown the chlamydial inclusion membrane protein CT135 to be an important virulence factor in the mouse genital tract model. These investigators isolated two C. trachomatis serovar D strains, termed D/LC for late clearance of genital tract infection and D/EC for early clearance of genital tract infection. The plaque-cloned strains were isogenic apart from CT135. The D/LC strain produced infections with significantly greater burden and of much longer duration that resulted in salpingitis compared to the D/EC strain. Interestingly, when C. trachomatis strains possessing both plasmid and CT135 virulence factors were evaluated together and independently in the mouse model, it was found that a strain deficient in both the plasmid and CT135 was more attenuated (18), demonstrating that both genetic loci were important to pathogenesis in the mouse. Because of these findings and those of Qu et al. (16), who showed minimal attenuation of their plasmid-deficient serovar D strain, we felt it important to perform a direct study in the female pig-tailed macaque model (19–23). We used a serovar D challenge strain that possessed both CT135 and plasmid (termed D/LC/P^+^) and a strain deficient in both CT135 and plasmid (termed D/EC/P^−^) to better define their roles in pathogenesis and potential for the development of a live-attenuated vaccine. We report no differences in the infectivities or pathogeneses of the D/LC/P^+^ and D/EC/P^−^ strains in pig-tailed macaques, findings like those reported by Qu at el. using rhesus macaques (16). However, in the present study we are the first to report elevated levels of the interleukin 1 (IL-1) receptor agonist (IL-1RA) in cervicovaginal secretions of infected macaques. We propose that IL-1RA might be the reason for the suppression of local genital tract inflammation in C. trachomatis-infected macaques. Thus, epigenetic innate immune host factors are likely more important than chlamydial virulence factors in determining the pathogenic outcome of chlamydial infection in the macaque female genital tract model.

(Findings from this study were presented, in part, at the World 2015 STI and HIV Congress, Brisbane, Australia, September 2015.)

## RESULTS

### Infection in macaques following primary cervicovaginal infection with D/LC/P^+^ and D/EC/P^−^ strains.

**FIG 1:**
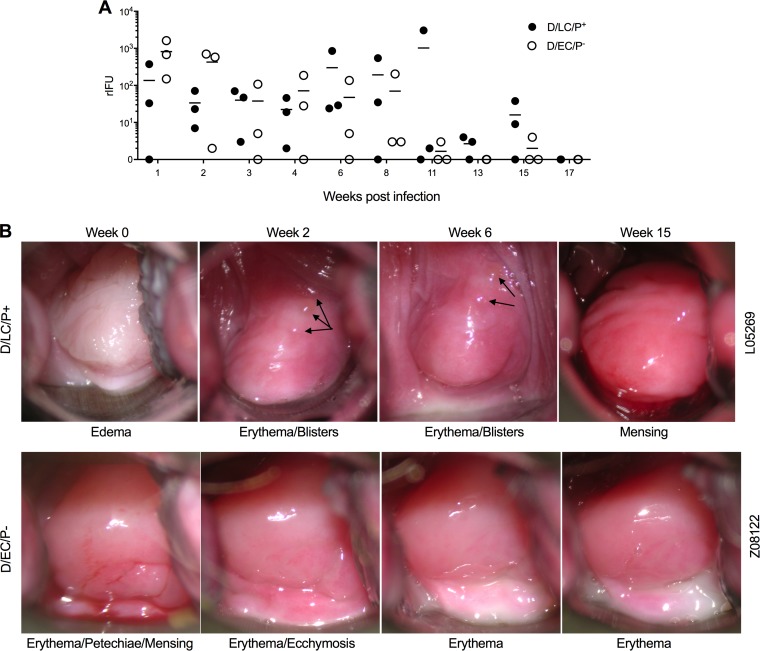
Infection and lower genital tract colposcopic pathological findings following primary cervicovaginal infection of macaques with the D/LC/P^+^ and D/EC/P^−^ strains. (A) rIFU recovered from cervical swabs from individual animals infected with D/LC/P^+^ and D/EC/P^−^. (B) Colposcopic images of the cervix from a representative animal infected with D/LC/P^+^ and D/EC/P^−^. General pathological findings for each animal at different weeks postinfection are given under the individual images. Only minor pathological changes in the cervix were observed, and they did not significantly differ between the infecting chlamydial strains. Arrows identify cervical blisters. Statistical analysis showed no significant difference in rIFU between groups.

The primary infection kinetics determined by infectious burden, infection duration, and gross clinical findings following colposcopy of three animals infected with the D/LC/P^+^ and D/EC/P^−^ strains is shown in Fig. 1. The infectious burden (recoverable inclusion-forming units [rIFU]) and infection duration did not differ significantly between groups of infected macaques (Fig. 1A). The infectious burden ranged between 10^1^ and 10^2^ rIFU for each weekly culture period for approximately 9 weeks postinfection (p.i.). Infectious loads decreased in both D/LC/P^+^- and D/EC/P^−^-infected animals at week 11, and by week 17 p.i., all animals had spontaneously cleared infection. There was a trend for D/LC/P^+^-infected animals to have higher rIFU from week 6 to 17, but this difference was not significant. Like for the culture results, we found no substantial differences in tissue inflammation between the D/LC/P^+^ and D/EC/P^−^ infections following colposcopic or laparoscopic examinations. One animal in the D/LC/P^+^ group (L05269) developed blisters on the cervix at weeks 2 and 6 (Fig. 1B).

### Gross pathology following primary infection.

**FIG 2:**
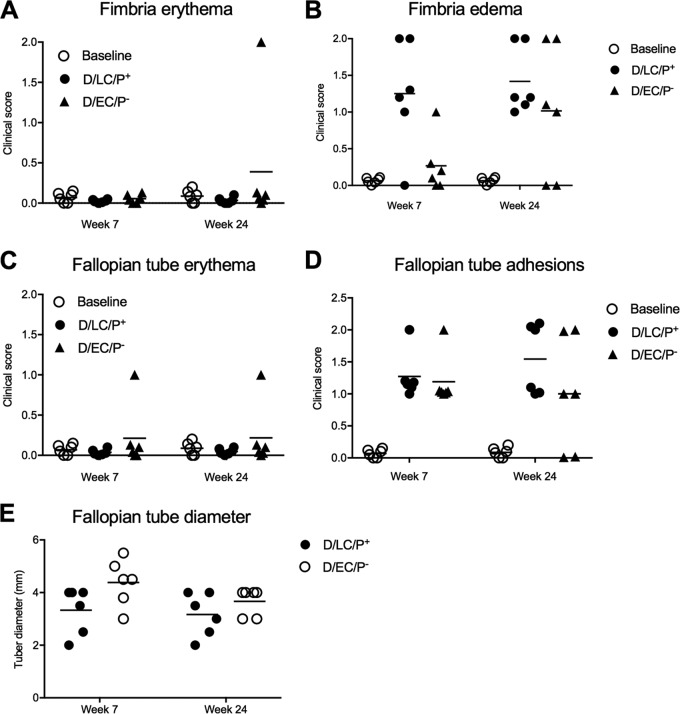
Upper genital tract laparoscopic pathological findings following primary cervicovaginal infection of macaques with the D/LC/P^+^ and D/EC/P^−^ strains. Gross pathological scores of animals obtained following scheduled laparoscopies compared to baseline values are shown. (A) Fimbria erythema; (B) fimbria edema; (C) fallopian tube erythema; (D) fallopian tube adhesions; (E) fallopian tube diameter.

Laparoscopy performed at weeks 7 and 24 p.i. revealed similar upper genital tract pathology among the two groups of animals (Fig. 2). These findings were unexpected because of the distinct differences in pathogenicity between the D/LC and D/EC strains in mice. D/LC produced infections with greater burden and of longer duration that resulted in salpingitis (17).

### Immune response following primary infection.

**FIG 3:**
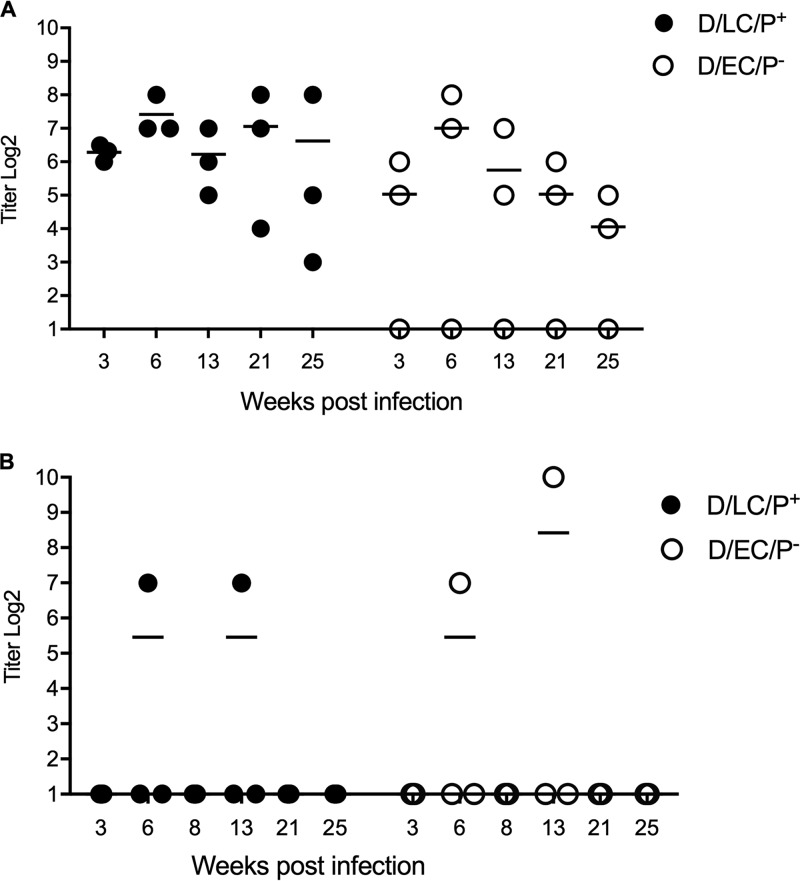
Serum and local antibody responses following primary cervicovaginal infection of macaques with the D/LC/P^+^ and D/EC/P^−^ strains. (A) Serum IgG antibody; (B) cervical IgG antibody. IgA was not detected in sera or cervical secretions.

Three macaques in the D/LC/P^+^ group and two macaques in the D/EC/P^−^ group developed serum antibody after the initial cervical challenge (Fig. 3). Serum IgG titers ranged from 1:32 to 1:128 and were positive at week 3 p.i. and through week 25. There was a trend for IgG titers to be higher in D/LC/P^+^-infected animals. Serum antichlamydial IgA was not detected for any animal in either group. Local cervicovaginal wash IgG antibody was detected in one animal of each group both at days 6 and 13 p.i. No antichlamydial IgA was detected in the cervicovaginal washes of any infected animal.

### Culture and serology of multiply rechallenged macaques.

**FIG 4:**
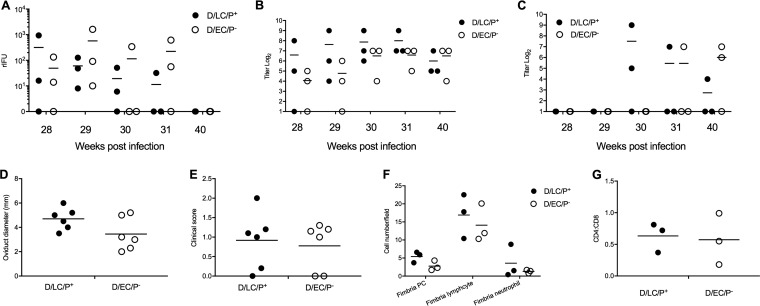
Infection, upper genital tract pathology, histopathology, and immunohistochemistry of macaques given multiple rechallenges with the D/LC/P^+^ and D/EC/P^−^ strains. Data for individual animals infected with D/LC/P^+^ and D/EC/P^−^ are shown in each panel. (A) rIFU. (B) Serum IgG antibody. (C) Local IgG antibody. (D) Oviduct diameters. (E) Clinical scores of oviduct adhesions. (F) Cell numbers of fimbria PC, fimbria lymphocytes, and fimbria neutrophils. (G) CD4/CD8 T cell ration in fimbriae. Statistical analysis showed no significant difference in rIFU between groups.

Most animals in both groups were infected following each successive challenge dose and shed similar numbers of rIFU postchallenge, indicating that primary infection or reinfection did not confer significant protective immunity to rechallenge (Fig. 4A). Serum antichlamydial IgG was detected in animals of both groups, and the titers remained relatively unchanged from that found following the primary challenge (Fig. 4B). Similarly, local IgG antibodies were either negative or at very low titer (Fig. 4C).

### Repeated cervicovaginal rechallenge with D/LC/P^+^ and D/EC/P^−^ strains results in minor inflammatory upper genital tract changes.

**FIG 5:**
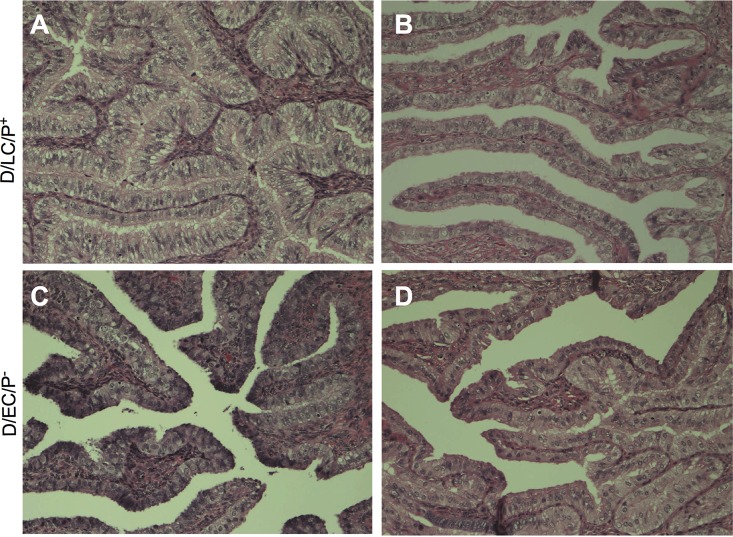
Hematoxylin and eosin staining of fallopian tubes following multiple rechallenges with the D/LC/P^+^ and D/EC/P^−^ strains. (A and B) Representative images of the upper genital tracts of two macaques infected with D/LC/P^+^, showing minimal to mild salpingitis and diffuse moderate epithelial cell hyperplasia. (C and D) Representative images of the upper genital tracts of two macaques infected with D/EC/P^−^, showing mild chronic salpingitis. Magnification, ×200 for all photographs.

Histopathologic examination of the reproductive tract, liver, and mesenteric and inguinal lymph nodes was performed on all animals after complete postmortem examinations. Interpretations of the histology of the reproductive tract ranged from those consistent with normal background findings in the mucosa of mature, hormonally cycling pig-tailed macaques (histologic score of 0). Histologic changes were compared to a subset of mature female pig-tailed macaques that were not exposed to C. trachomatis. Animals in both rechallenged infected groups showed small numbers of predominantly small lymphocytes seen throughout the superficial submucosa and within the surface epithelial layers, with fewer macrophages and plasma cells (PC) and very rare neutrophils, interpreted as background inflammation (Fig. 5). There were no differences in oviduct dilation/adhesins (Fig. 4D and E) or fimbria PC, lymphocytes, or neutrophils (Fig. 4F). Immunohistochemical stains for CD4, CD8, CD20, CD68, KK12 (a C. trachomatis species-specific stain), and cHSP60 (chlamydia heat shock protein) were applied. No positive chlamydial inclusions were detected in stained sections. CD8^+^, CD4^+^ lymphocytes, neutrophils, and PC were detected within the upper reproductive tract, but the numbers did not differ among animals of the two infection groups (Fig. 4G).

### Cytokine profiles of D/LC/P^+^- and D/EC/P^−^-infected macaques.

**FIG 6:**
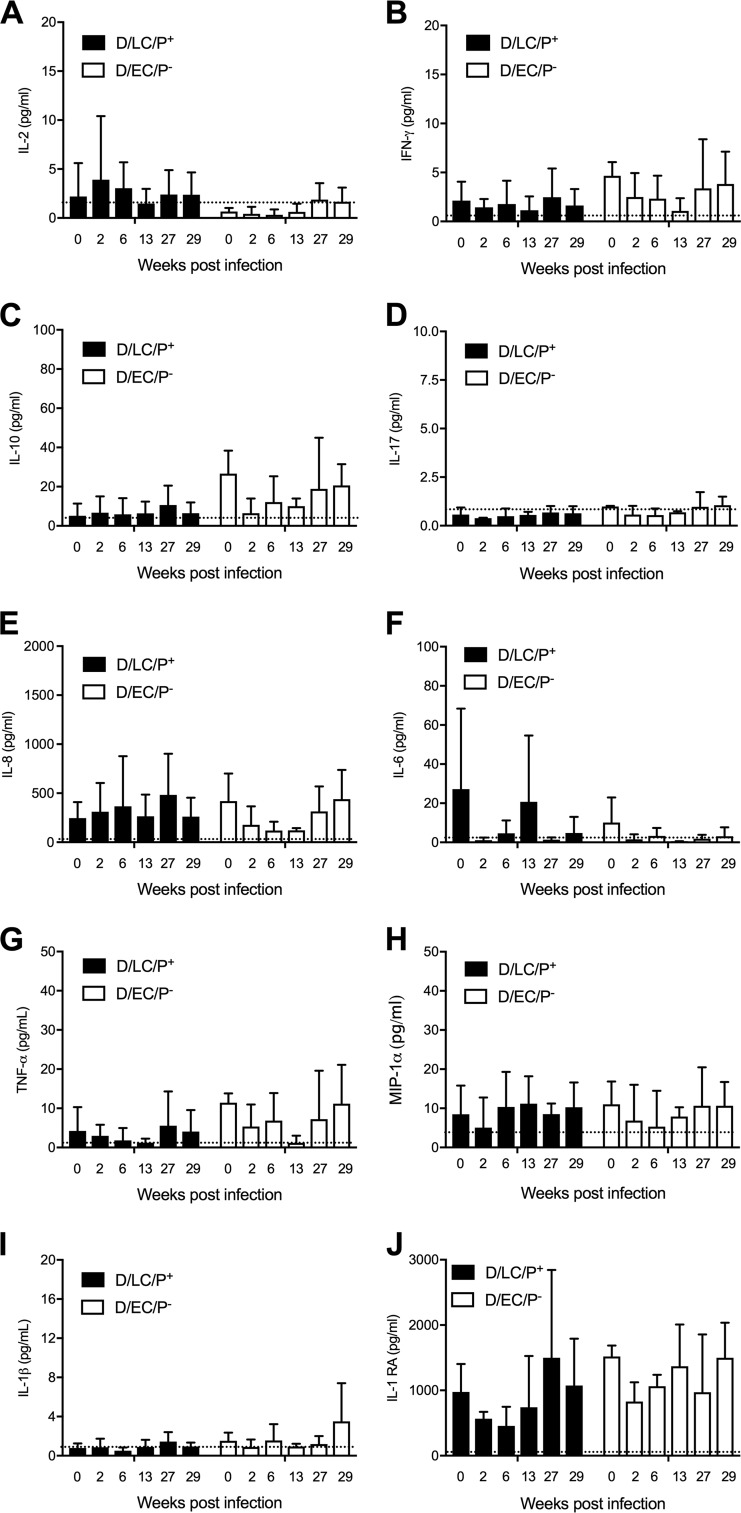
Cytokine analysis of cervical secretions following primary and multiple rechallenges with the D/LC/P^+^ and D/EC/P^−^ strains. (A) IL-2; (B) IFN-γ; (C) IL-10; (D) IL-17; (E) IL-8; (F) IL-6; (G) TNF-α; (H) MIP-1α; (I) IL-1β; (J) IL-1RA. Noteworthy is the very small amounts of IL-1β and elevated amounts of IL-1RA found in local cervical secretions in both D/LC/P^+^- and D/EC/P^−^-infected animals prior to challenge and throughout the primary- and multiple-challenge periods. The dotted line in each of the panels represents the minimal amount of cytokine detected for individual assays. Statistical analysis showed no significant difference between groups.

Cervicovaginal secretions were assayed for different cytokines throughout the primary and rechallenge study periods (Fig. 6). The cytokine assayed were IL-2, gamma interferon (IFN-γ), IL-10, IL-17, IL-6, IL-8, tumor necrosis factor alpha (TNF-α), MIP-1α, IL-1β, and IL-1RA. The results are shown in Fig. 6. There were no significant differences in either the type or concentration of these cytokines between infection groups. Notably, macaques infected with either challenge strain had very small amounts of IL-1β in cervicovaginal secretions (Fig. 6I), a finding consistent with the minimal inflammatory pathology evoked following primary or repeated challenge infections. Interestingly, we found very high concentrations (1,000 to 2,000 pg/ml) of the IL-1β receptor agonist, IL-1RA, in both preinfection (day 0) and postinfection secretions that remained elevated throughout the primary and secondary infection challenges (Fig. 6J). The balance between IL-1 and IL-1RA in local tissues plays a significant role in the susceptibility to and severity of many diseases (24). These findings in part provide a logical explanation for the dampened inflammatory response observed in macaques despite repeated attempts to drive genital tract pathology.

## DISCUSSION

Previous studies using mouse and macaque animal models have identified the plasmid and CT135, an inclusion membrane protein, as important virulence factors. Plasmid-free C. muridarum and C. trachomatis LGV strains are partially attenuated for the mouse genital tract (8, 10, 25, 26). Plasmid-free trachoma organisms are highly attenuated in the cynomolgus macaque model for ocular infection (9). In addition to the plasmid, C. trachomatis CT135 has also been identified as an important virulence factor in the female mouse model (17), and in the mouse model it has a more dominant effect on pathogenicity than the plasmid (18). Despite these important virulence traits, the molecular mechanisms by which the plasmid and CT135 confer virulence to chlamydial pathobiology and how host factors contribute to these characteristics are not completely understood. As both the plasmid and CT135 are important to the pathobiology of C. trachomatis infection, we felt that it was important to study the effects of both plasmid and CT135 in the macaque female genital tract model. To this end, we infected macaques with isogenic serovar D strains differing in these two virulence factors. Our findings show that neither the plasmid nor CT135 plays a dominant role in the pathogenesis of C. trachomatis female genital infection in nonhuman primates. Only a modest difference was noted in chlamydial shedding and disease progression between D/LC/P^+^- and D/EC/P^−^-infected animals. Blinded upper genital tract gross laparoscopic pathology scores resulted in similar findings. Histopathologic findings correlated with the degree of severity of the gross findings at necropsy and yielded no discernible differences between the P^+^- and P^−^-infected groups. Our findings agree with those reported by Qu et al. (16), who also concluded that the plasmid of a human urogenital strain (serovar D) does not play a dominant role in the pathogenesis using a female rhesus macaque infection model.

The clear discrepancies between the results obtained using the plasmid-free C. trachomatis strain (9) and those reported here and by Qu et al. (16) are not understood completely, but there are plasmid-independent genetic differences between the ocular and genital strains that might account for the differences in infection and pathogenic outcomes. First, the highly attenuated C. trachomatis A2497P^−^ strain has disruptions in several chromosomal genes that are not found in urogenital strains. TrpA, which confers resistance to the inhibitory effect of IFN-γ-mediated tryptophan starvation in epithelial cells (14), and CT166, a putative cytotoxin (13) of unknown function in chlamydial pathogenesis, are disrupted in ocular but not urogenital strains (27). Intuitively, these differences implicate the genes as encoding important virulence factors in urogenital strains that could compensate for plasmid virulence factor function(s). Second, the plasmid-independent virulence gene CT135 (17) in D/LC, D/EC, and the ocular A2497 strain has frameshift mutations differing from that of the initially annotated serovar D strain (28). The frameshift in serovar A and D/EC strains disrupts CT135 near the middle of the gene, generating two similarly sized, smaller proteins. In contrast, the frameshift in D/LC results in a slightly shorter predicted CT135 protein that could maintain, at least in part, its function. That the D/LC CT135 open reading frame (ORF) retains some functional activity is supported by the greater pathogenicity of this strain for the mouse genital tract (17). However, these CT135 mutations did not similarly attenuate the urogenital D strain in the macaque female genital tract infection model. We speculate that the mutation in the D/LC CT135 gene might have a more powerful attenuating phenotype in mice than in nonhuman primates. Interestingly, Borges et al. (29) described deletion events involving CT135 that impacted the expression of plasmid-independent virulence-associated genes involved in host cell invasion and immune subversion. Thus, it is possible that both the plasmid and CT135 function in the regulation of chlamydial virulence genes. This scenario, depending on the genetic background of individual chlamydial strains, could affect infection outcomes in a host- or organ-specific manner. This possibility should be considered in the design of future animal model experiments that target the plasmid and CT135 as attenuating virulence factors. Clearly, to fully understand the role and importance of CT135 in the pathogenesis of chlamydial infection, it will be essential to study clonal strains with an intact CT135 ORF separately and in P^+^ and P^−^ strain backgrounds. A cautionary note in this regard is that although the CT135 ORF is consistently found to be intact in human clinical isolates (17, 30), it becomes disrupted at a very high frequency in strains that are sequentially passed in cell culture (17, 30, 31). The strong negative selection for CT135 *in vitro* requires that investigators confirm the genetic integrity of CT135 prior to doing *in vivo* pathogenesis studies. In the future, it will be very important to understand the genetic and epigenetic differences among ocular and genital P^+^ and P^−^ strains to fully appreciate the plasmid's role in pathogenesis and to develop highly attenuated strains that can be used as safe and efficacious vaccines. That said, it is the responsibility of investigators to understand how both the genetic and epigenetic factors discussed here might influence experimental outcomes that could be both strain and host dependent.

Although we did not find significant differences in local cytokine profiles between D/LC/P^+^- and D/EC/P^−^-infected macaques, we did observe an inverse correlation between the lack of inflammatory pathology and the levels of IL-1β and IL-1RA in cervical secretions (Fig. 6I and J). In fact, this inverse relationship between an inflammatory cytokine and its agonist was present in all animals prior to infection. This raises the interesting question of whether this epigenetic host factor might be a reason that macaques are not particularly susceptible to inflammatory disease following aggressive C. trachomatis infection challenges, as it may require multiple infectious challenges to overcome the elevated levels endogenous IL-1RA. These observations contrast with murine (32) and guinea pig (33) models of female genital tract infection, where primary single genital tract infection with host-specific strains is sufficient to induce ascending infection with severe upper genital tract inflammatory pathology. Moreover, in both aforementioned models, infection elicits protective immunity. In the mouse model, protective immunity is mediated by CD4^+^ T cells (34, 35). Interestingly, it has been shown that IL-1β signaling in T cells markedly induces robust and durable CD4^+^ responses (36). Collectively, these observations may help explain the poor protective immunity against C. trachomatis genital infection observed in macaques. It would be interesting to know if there is a similar correlation in the human immune response to C. trachomatis infection. Hvid et al. (37) showed that infection with C. trachomatis and treatment with IL-1RA eliminated tissue destruction in human fallopian tube cultures. Epidemiology studies have examined the potential role for IL-1 gene polymorphisms in chlamydial infection. Momiyama et al. (38) concluded that IL-1 gene polymorphisms played a role in the development of coronary artery disease associated with Chlamydia pneumoniae infection. Conversely, Murillo et al. (39) found no relationship between IL-1 gene polymorphisms and the severity of C. trachomatis genital infection; however, a caveat in their study was the relatively small study cohort. To our knowledge, there have been no reports examining the relative ratios of IL-1 and ILRA in relationship to the severity of human C. trachomatis infection and disease outcome. As asymptomatic chlamydial infections are quite common in humans, could an imbalance in the ratio, with IL-1RA > IL-1, be a possible epigenetic explanation for asymptomatic infection? Conversely, might the reciprocal imbalance, IL-1 > IL-1RA, be a risk factor for postinfection inflammatory sequelae in the upper genital tract? In summary, understanding the pathobiology of chlamydial infection in animal models and humans requires a greater understanding of both chlamydial and host genetic factors.

## MATERIALS AND METHODS

### Nonhuman primates.

Twelve sexually mature female pig-tailed macaques (Macaca nemestrina), age 4 to 13 years, were used in the study. All monkeys were housed at the Washington National Primate Research Center. Prior approval for use of monkeys in this protocol was obtained from the Institutional Animal Care and Use Committee at the University of Washington. The animals were monitored daily for 1 month after assignment for general health and to document normal menstrual cycles. The use and care of animals for this study followed the guidelines approved by the University of Washington Institutional Animal Care and Use Committee.

### Chlamydia trachomatis strains, cell culture propagation, and purification.

The wild-type C. trachomatis D/LC/P^+^ and plasmidless D/EC/P^−^ strains were both derived from a parental serovar D stock, D/UW-3/Cx. The D/EC/P^−^ strain was cured of its plasmid as previously described (18). Both strains were plaque cloned and propagated in McCoy or HeLa 229 cells, and elementary bodies were purified as described previously (40). The D/LC and EC strains are isogenic except in CT135, where each strain contains either a single-nucleotide T deletion or T insertion that results in frameshift disruption of the protein (17). In mice, the EC and LC strains exhibit early and late clearance kinetics, respectively, and the LC strain naturally ascends to the upper genital tract, causing salpingitis (17).

### Cervical infection of pig-tailed macaques.

**FIG 7:**
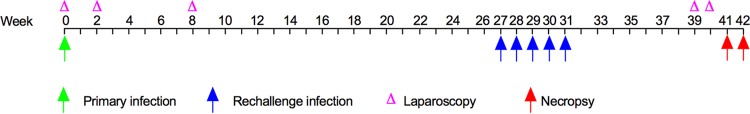
Schematic diagram of the design and experimental procedures used in this study. Twelve female pig-tailed macaques were inoculated cervicovaginally with 1 ml of sucrose-phosphate-glutamine containing 10^6^ IFU of C. trachomatis strains D/LC/P^+^ (*n* = 6) and D/EC/P^−^ (*n* = 6) at week zero. Macaques were repeatedly rechallenged with each strain at weekly intervals between week 27 and 31. Infection was followed weekly by culture and NAATs, testing of sera and cervical secretions for antichlamydial antibodies, and colposcopic examination for clinical lower genital tract disease. Laparoscopy was performed to evaluate upper genital tract pathology at baseline, at weeks 2 and 8 after primary infection, and at week 39 to 40 after rechallenges. Following primary challenge of six macaques for each chlamydial strain, only 3 animals in each group were culture positive. The 3 infected animals for each strain were followed for the remainder of the study. All animals were euthanized and necropsied at week 41 to 42.

Pig-tailed macaques underwent prebaseline assessments a minimum of 2 weeks prior to the first study visit. This prebaseline visit included collection of 3 ml of blood for determination of serum anti-C. trachomatis antibody to rule out previous chlamydial infection and a laparoscopy to confirm normal status of the upper reproductive tract tissues. The distal fallopian tube (fimbria and ampulla) tissues were assessed for color, size, flexibility, and absence of adhesions, and the surrounding peritoneum was assessed for ascites. Six animals were randomly assigned to each of two study groups: one group received C. trachomatis D/LC/P^+^, and the other group received D/EC/P^−^. The study design is illustrated in Fig. 7. Animals were inoculated cervicovaginally with 1 ml containing 1 × 10^6^ IFU of the appropriate C. trachomatis strain on day 0. For unknown reasons, only 3 of 6 animals in each group showed evidence of infection as determined by culture and nucleic acid amplification tests (NAATs); therefore, the findings reported here are restricted to these animals. Infected animals were followed weekly (weeks 1 to 8) for clinical evidence of infection, collection of cervical swabs for culture and NAAT, blood draws for serum antibody detection, and collection of sponge secretions for local antibody and cytokine/chemokine detection. Tissue inflammation of the lower reproductive tracts of infected animals was monitored by colposcopy weekly. The upper reproductive tract was monitored by blinded observers at laparoscopy to assess upper tract status after a single intravaginal inoculation and prior to the repeated weekly cervical challenges. At week 27, all animals had spontaneously resolved infection, i.e., were culture and NAAT negative. Each animal then received five weekly cervicovaginal challenges with matched strains (weeks 27 to 31) to promote upper tract disease. Animals were cultured weekly. Sera and local secretions were also collected weekly and analyzed for chlamydial antibodies. Laparoscopy was performed at week 39 to 40; final reproductive tract fimbrial swabs for C. trachomatis detection and tissue collections occurred at necropsy (week 41 to 42).

### Detection of Chlamydia trachomatis from cervical swabs.

Cervicovaginal swab specimens were cultured onto cycloheximide-treated McCoy cells in 24-well microtiter plates with a single blind pass and stained with monoclonal antibody specific for chlamydial lipopolysaccharide (41). The cultures were inoculated both at their original concentration and at a 1:10 dilution. The number of recoverable inclusion-forming units (rIFU) was determined after the first cell passage. An NAAT performed using the Aptima Combo 2 assay (Gen-Probe, Inc., San Diego, CA) was used to detect C. trachomatis RNA from cervical swabs per the manufacturer's instructions (42). The Aptima assay employs transcription-mediated amplification, in which the RNA target molecule from C. trachomatis (23S rRNA) is isolated and specific regions are amplified by using a separate capture oligomer and a unique set of primers for the target.

### Assessment of cervical and upper reproductive tract inflammation.

Colposcopy was performed to assess the cervix following the standardized observation guideline developed specifically for macaque studies (43). Tissues were evaluated for the presence of erythema, edema, petechia, friability, and/or blister formation. Laparoscopy was done to assess the upper reproductive tract (URT). The distal fallopian tube (fimbria and ampulla) was assessed for erythema, edema, flexibility, presence/absence of adhesions, and ascites. Gross tissue changes were scored as 0 (normal), 1+ (mild), 2+ (moderate), or 3+ (severe) for the lesions erythema, edema, and adhesion formation, and tubal diameter was directly measured.

### Antibody and cytokine detection.

Serum IgG and IgA chlamydial antibody titers were measured using the microimmunofluorescence technique (44). Cervical secretions were collected with Merocel sponges (Medtronic Ophthalmics, Minneapolis, MN, USA) throughout the study for local antibody and cytokine detection. The sponge was placed at the cervical os and allowed to absorb secretions for 2 min. The secretions were extracted using the protocol described by Castle et al. (45). The secretions were divided into two equal aliquots. One aliquot of the genital secretion eluates was assessed for cytokine/chemokine detection using a Luminex assay format. The cytokines assayed were IL-2, IFN-γ, IL-10, IL-17, IL-6, IL-8, TNF-α, MIP-1α, IL-1β, and IL-1RΑ. The second aliquot was used to detect local anti-C. trachomatis IgA and IgG.

### Histologic and immunohistochemical analyses of genital tract tissue.

Tissues from the lower and upper reproductive tracts of each monkey were stained by hematoxylin and eosin for routine histologic assessments of polymorphonuclear cells, lymphocytes, and plasma cells (PC), which were quantified by averaging their numbers in 5 nonadjacent high-power fields (hpf) (magnification, ×400). Histopathology was scored by two investigators (A.E.B. and D.L.P.) who were blinded to the infection status of the macaques. Immunohistochemical staining of immune cell subsets included CD68, CD4^+^, CD8^+^, and CD20 cells. They were quantified by averaging 5 nonadjacent hpf (magnification, ×400). Serial sections from paraffin-embedded tissues were incubated with C. trachomatis-species specific monoclonal antibody KK12, which recognizes the 40-kDa major outer membrane protein (MOMP), or with antibody to chlamydial HSP60.

### Statistical analysis.

Results were analyzed using GraphPad Prism v7 and are presented as the arithmetic mean values. The significance of differences was determined using a two-sample *t* test with the statistical R program.
